# Nano Zinc Oxide Green-Synthesized from *Plumbago auriculata* Lam. Alcoholic Extract

**DOI:** 10.3390/plants10112447

**Published:** 2021-11-12

**Authors:** Mina Michael Melk, Seham S. El-Hawary, Farouk Rasmy Melek, Dalia Osama Saleh, Omar M. Ali, Mohamed A. El Raey, Nabil Mohamed Selim

**Affiliations:** 1 Pharmacognosy Department, Faculty of Pharmacy, Cairo University, Giza 12613, Egypt; dr.mina.michel@gmail.com (M.M.M.); seham.elhawary@yahoo.com (S.S.E.-H.); 2 Chemistry of Natural Compounds Department, National Research Centre, Giza 12622, Egypt; frmelek1@gmail.com; 3 Pharmacology Department, National Research Centre, Giza 12622, Egypt; doabdelfatah@yahoo.com; 4 Department of chemistry, Turabah University College, Turabah Branch, Taif University, P.O. Box 11099, Taif 21944, Saudi Arabia; 5 Department of Phytochemistry and Plant Systematics, Pharmaceutical Division, National Research Centre, 33 El Bohouth Street, P.O. Box 12622, Dokki, Cairo 12622, Egypt; elraiy@gmail.com

**Keywords:** ZnO NPs, *Plumbago auriculata* Lam., HPLC analysis, antiviral activity, green synthesis

## Abstract

Zinc oxide nanoparticles (ZnO NPs) were synthesized by using an alcoholic extract of the flowering aerial parts of *Plumbago auriculata* Lam. Dynamic Light Scattering (DLS) revealed that the average size of synthesized ZnO NPs was 10.58 ± 3.350 nm and the zeta potential was −19.6 mV. Transmission electron microscopy (TEM) revealed that the particle size was in the range from 5.08 to 6.56 nm. X-ray diffraction (XRD) analysis verified the existence of pure hexagonal shaped crystals of ZnO nanoparticles with an average size of 35.34 nm in the sample, which is similar to the particle size analysis acquired by scanning electron microscopy (SEM) (38.29 ± 6.88 nm). HPLC analysis of the phenolic ingredients present in the plant extract showed that gallic acid, chlorogenic acid, and catechin were found as major compounds at concentrations of 1720.26, 1600.42, and 840.20 µg/g, respectively. Furthermore, the inhibitory effects of ZnO NPs and the plant extract against avian metapneumovirus (aMPV) subtype B were also investigated. This assessment revealed that the uncalcinated form of Nano-ZnO mediated by *P. auriculata* Lam. extract possessed a significant antiviral activity with 50% cytotoxic concentration (CC_50_) and 50% inhibition concentration (IC_50_) of 52.48 ± 1.57 and 42.67 ± 4.08 µg/mL, respectively, while the inhibition percentage (IP) was 99% and the selectivity index (SI) was 1.23.

## 1. Introduction

Utilization of inorganic nanoparticles (NPs) for various applications, and consideration of environmental aspects has stimulated demand for synthesizing them via green chemistry approaches. Plants have become the preferred candidates for nanoparticle biosynthesis at large scales due to their rapid synthesis rates and the variations of the shape and size of the produced nanoparticles. Furthermore, many bioactive constituents of plants such as alkaloids, terpenoids, flavonoids, enzymes, amino acids, proteins, vitamins, and glycosides could be responsible for bio reduction, creation and preservation of metal nanoparticles [1].

*Plumbago auriculata* Lam. is a shrub native to South Africa and successfully acclimatized in tropical and subtropical regions of the world. This plant is referred to as Cape Leadwort or Cape *Plumbago* because it is abundant in the Cape districts of South Africa. The plant has the ability to adapt to various stressful conditions such as high humidity, high temperature, and diseases. *P. auriculata* has been recognized as a medicinal plant as it possesses numerous potent bioactive constituents such as α-amyrin, α-amyrin acetate, capensisone, isoshinanolene, *β*-sitosterol, plumbagin, isoshinanolone, diomuscinone, diomuscinone, and *β*-sitosterol-3-*β*-glucoside [2].

Several metal NPs have been successfully biosynthesized from different *Plumbago* species. Sliver NPs were previously green-synthesized using various *Plumbago* species such as *P. indica*, *P. auriculata*, and *P. zeylanica.* The produced silver NPs possessed an antioxidant activity and antitumor activity against Dalton’s Lymphoma as well as antibacterial activity against Gram-positive and Gram-negative bacteria such as *Bacillus subtilis*, *Staphylococcus aureus*, *Escherichia coli,* and *Klebsiella pneumonia.* They also showed larvicidal activity against *Aedes aegypti* and *Culex quinquefasciatus* and antitubercular activity against *Mycobacterium tuberculosis* [3,4,5,6]. Selenium NPs were previously synthesized by green methods using *P. zeylanica* extract and found to have an antioxidant and antibacterial activities [7]. Copper NPs were successfully synthesized using *P. zeylanica* and were proven to be an antidiabetic nanomedicine [8]. Silver, gold, and bimetallic (silver and gold) NPs were produced from the medicinal plant *P. zeylanica* and showed antimicrobial and antibiofilm activities against *Escherichia coli,* *Acinetobacter baumannii,* *Staphylococcus* *aureus,* and a mixed culture of *Acinetobacter baumannii* and *Staphylococcus* *aureus* [9]. Highly stable and spherical zinc oxide NPs were also produced using *P. zeylanica* leaf extract and were found to have antibacterial activity against *Staphylococcus aureus and Salmonella typhimurium* [10].

Nanotechnology has extensively expanded in virology fields for various applications such as prophylactic, therapeutic, and diagnostic approaches. Nanoparticles have been applied for imaging purposes and as an adjuvant carrier for drugs to augment virucidal properties. Antiviral NPs have mainly been applied against hepatitis virus types A, B, C, and E, human immunodeficiency virus (HIV), and herpes simplex virus (HSV-1 and HSV-2) [11]. Nano-ZnO which allows zinc to be absorbed easily by the body, was categorized as a “GRAS” (generally recognized as safe) substance by the FDA (US Food and Drug Administration). Compared with other metal oxide NPs, ZnO NPs are considered to be an inexpensive and less toxic material and to exert a noticeable effect in several biomedical applications such as anticancer, drug delivery, antimicrobial, antidiabetic, anti-inflammatory, wound healing, and bio-imaging. [12]

Avian metapneumovirus (aMPV) is a single-stranded RNA (negative-sense) enveloped virus belonging to the Metapneumovirus genus of the Paramyxoviridae family. This family comprised viruses responsible for producing respiratory ailments in humans and animals. In chickens, the aMPV is responsible for the incidence of a multi-factorial disease identified as the swollen head syndrome (SHS) [13]. Good management practices and rigorous biosecurity play key roles in avoidance of infection as well as minimizing the effects of an aMPV infection. There is no treatment available for aMPV infections; however, aMPV infections can be prevented by vaccination. Attenuated vaccines have been applied to control infections in growing turkeys and broiler chickens and facilitate the injection of inactivated vaccines for future layers and breeders before the onset of lay [14].

Most studies have been dedicated to the inhibition activity of ZnO NPs on bacterial infections, however there are few works focused on evaluating the interaction between *P. auriculata* or ZnO NPs and viruses.

The current research was designed to scrutinize the antiviral activities of the *P. auriculata* ethanolic extract and its ZnO NPs on aMPV, which is considered to be one of the most challenging viruses causing significant economic losses in poultry cultivation.

## 2. Results and Discussion

### 2.1. HPLC Investigation of Phenolic and Flavonoid Compounds

**Figure 1:**
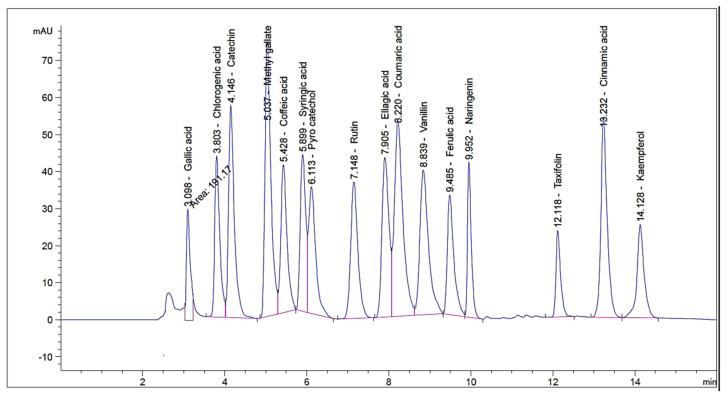
HPLC chromatogram of phenolic compounds (standard mixture).

**Figure 2:**
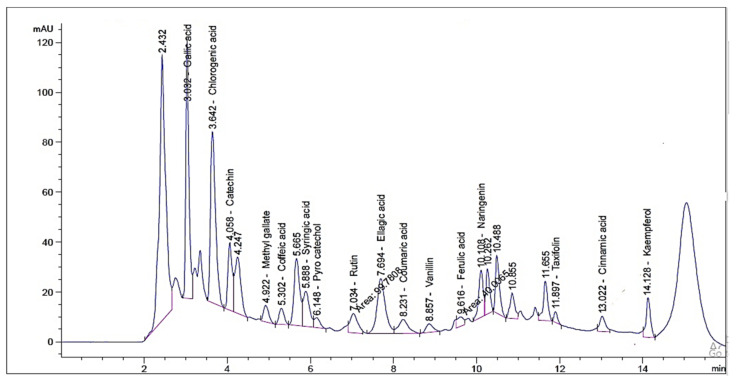
HPLC chromatogram of *P. auriculata* flowering aerial parts (ethanolic extract).

HPLC investigation revealed the occurrence of sixteen compounds in the alcoholic extract of flowering aerial parts of *P. auriculata* Lam. Gallic acid, chlorogenic acid and catechin were found to be major components at concentrations of 1720.26, 1600.42, and 840.20 µg/g respectively (Table 1). The detection of taxifolin was previously reported by Skaar et al. [15] who isolated taxifolin glycoside from the plant flowers. The occurrence of the rest of the identified compounds is presented here for the first time. However, the previous studies informed the isolation of the phenolic compounds apigenin, capensinidin, europinidin, pulchellidin, azalein, and capensinidin-3-rhamnoside from the flowers [16,17], together with apigenin, luteolin, and their glycosides from the leaves [17]. By reviewing the current literature, this is the first analysis for identification of phenolic compounds in *P. auriculata* by using HPLC methodology (Figure 1 and Figure 2).

### 2.2. Characterization of ZnO Nanoparticles

#### 2.2.1. UV Analysis

**Figure 3:**
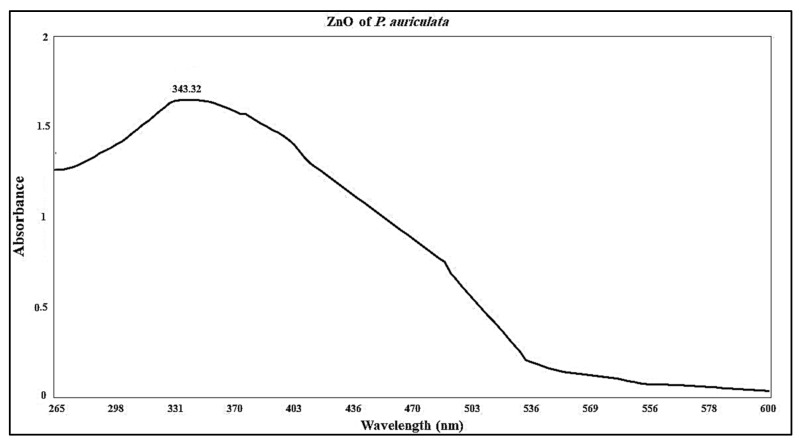
UV spectrum of biosynthesized ZnO nanoparticles.

Nano-ZnO produced through *P. auriculata* alcoholic extract showed a maximum absorption peak at 343.32 nm, indicating the synthesis of Nano-ZnO (Figure 3) [18], since ZnO on a nanoscale has absorption patterns at wavelengths shorter than conventional ZnO. This is in agreement with pervious findings which showed shorter wavelengths for the nanoparticles of material oxides [19].

#### 2.2.2. FT-IR Analysis of Nano-ZnO and *P. auriculata* Lam.

**Figure 4:**
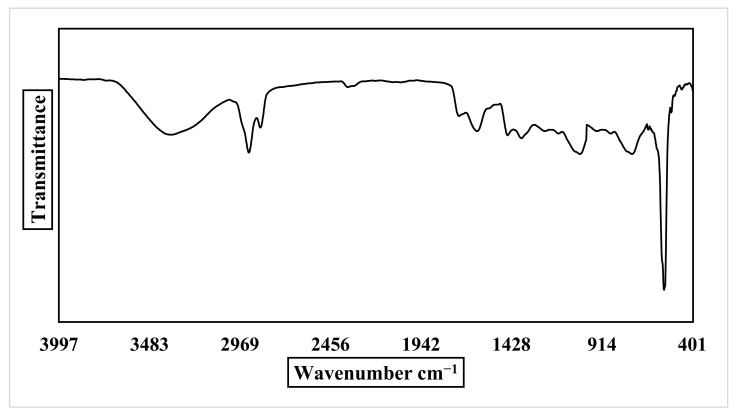
FTIR spectrum of ZnO NPs.

**Figure 5:**
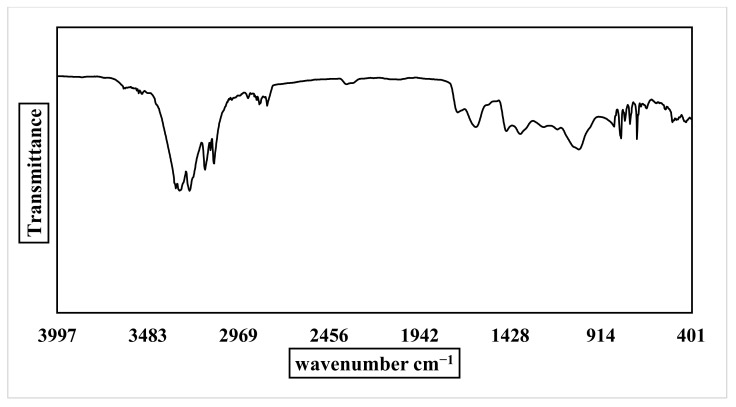
FTIR spectrum of the alcoholic extract of the aerial parts of *P. auriculate*.

The FTIR spectra were examined in the range from 400 to 4000 cm^−1^ to identify the different reactant groups involved in ZnO NPs synthesis and *P. auriculata* extract. According to the ZnO NPs spectrum (Figure 4), an intense absorption band at 3372 cm^−1^ (OH) was observed and attributed to water adsorption on the ZnO NPs surface. The bands at 2919 and 2850 cm^−1^ are due to CH stretching. Absorption bands at 1724, 1617, and 1449 cm^−1^ indicate C=O stretching of carboxylic group. The existence of a stretch band at 1032 cm^−1^ is due to CO, revealing the presence of the active groups for alcohols, ethers, and carboxylic acid esters. The emergence of new peak at 744 confirms that the ZnO NPs underwent CH bending, and the characteristic band of the ZnO NPs stretching mode was assigned to 561 cm^−1^. FT-IR results for *P. auriculata* extract (Figure 5) presented several bands at 3311, 3302, and 3245 cm^−1^ confirming the presence of an OH group in phenolic and alcoholic compounds. Medium peaks found at 3158, 3128, 3109, 2916, 2844, and 2804 cm^−1^ characterized the existence of a stretch alkane group (CH). The bands at 1724, 1619, 1449, and 1371 cm^−1^ in the spectrum of the plant extract were due to the presence of CH bending vibrations and C–C (in–cycle) or C=O stretch in the phenolic constituents. The frequencies of these bands decreased in intensity in case of ZnO NPs due to the attachment of phenolic and amino groups. The peak acquired at 1036 cm^−1^ illustrated the existence of CH alkanes group. Medium bands obtained at 799, 777, 748, and 709 cm^−1^ signified =CH bending frequencies of an alkene group [20,21].

#### 2.2.3. DLS and Zeta Potential

**Figure 6:**
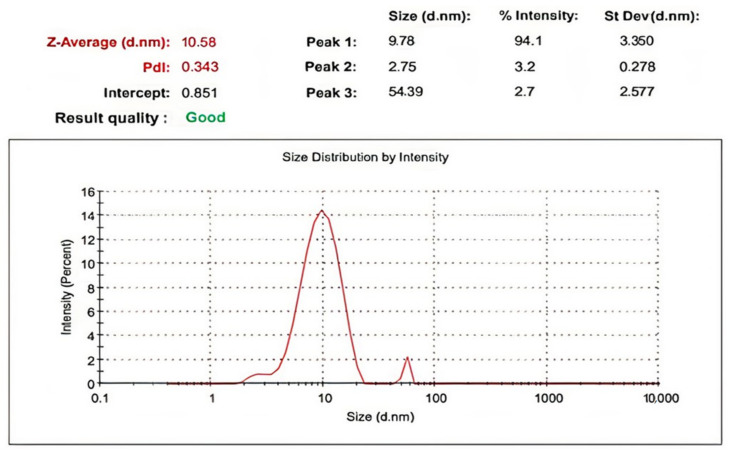
Zeta size of biosynthesized zinc oxide nanoparticles.

**Figure 7:**
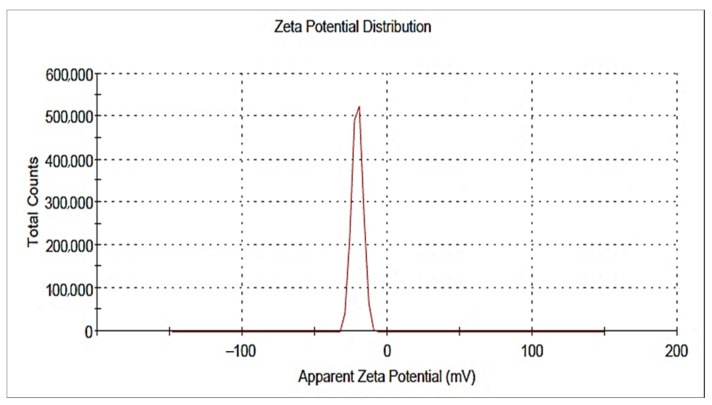
Zeta potential of biosynthesized zinc oxide nanoparticles.

A size-distribution image (DLS) of the green-synthesized form of Nano-ZnO is represented in Figure 6. The detected distribution of ZnO NPs size varied from 2 to 68 nm. The average particle size distribution of NPs was 10.58 ± 3.350 nm with a PDI value of 0.343. The ZnO NPs’ zeta potential was demonstrated to have a peak at −19.6 mV (Figure 7), signifying that the Nano-ZnO particles biosynthesized were negatively charged and moderately distributed in the medium. Negative values detected by zeta potential were responsible for stabilization of the nanoparticles.

#### 2.2.4. Transmission Electron Microscopy (TEM) and Scanning Electron Microscopy (SEM) Analyses

**Figure 8:**
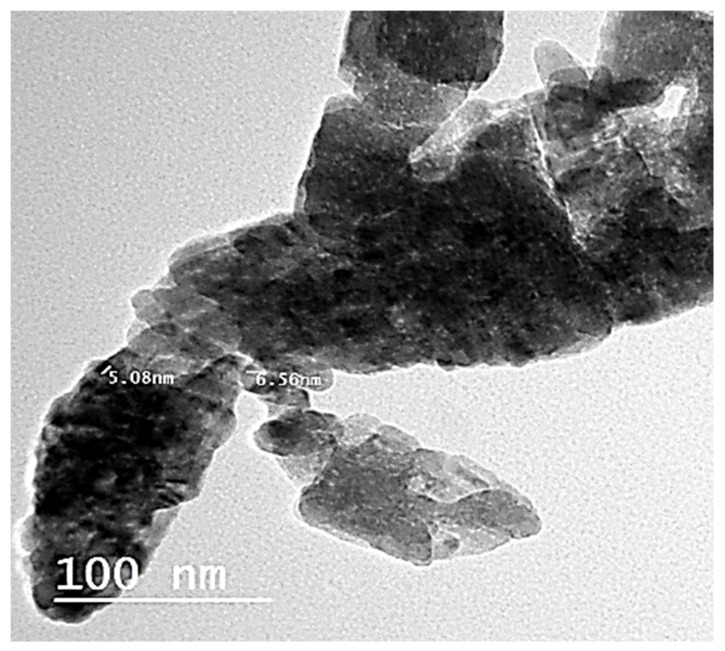
TEM analysis of ZnO NPs.

**Figure 9:**
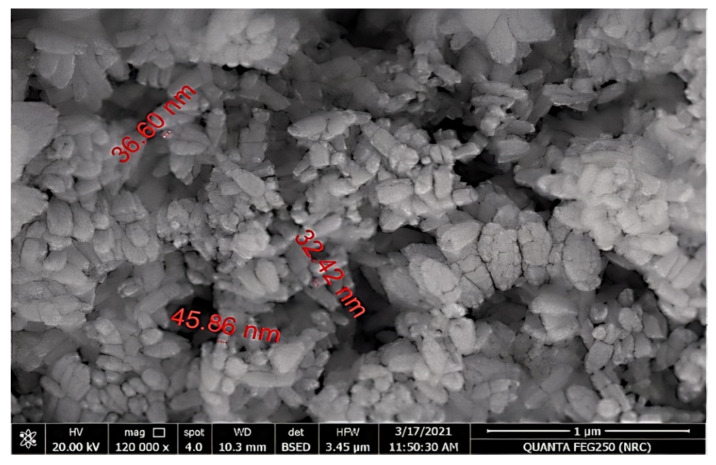
SEM analysis of ZnO NPs.

TEM examination using low- and high-resolution power revealed the production of hexagonal Nano-ZnO with particle sizes between 5.08 and 6.56 nm with an average size of 5.82 ± 0.74 nm, as shown in Figure 8. The image obtained for the Nano-ZnO surface morphology was inspected by SEM. It showed that most of the Nano-ZnO particles were spherical with some agglomerated in shape and the particle diameter was ranged from 32.42 to 45.86 nm with an average size of 38.29 ± 6.88 nm (Figure 9). SEM analysis illustrated the morphology and size of Nano-ZnO that was dispersed moderately in the medium.

#### 2.2.5. XRD Analysis

**Figure 10:**
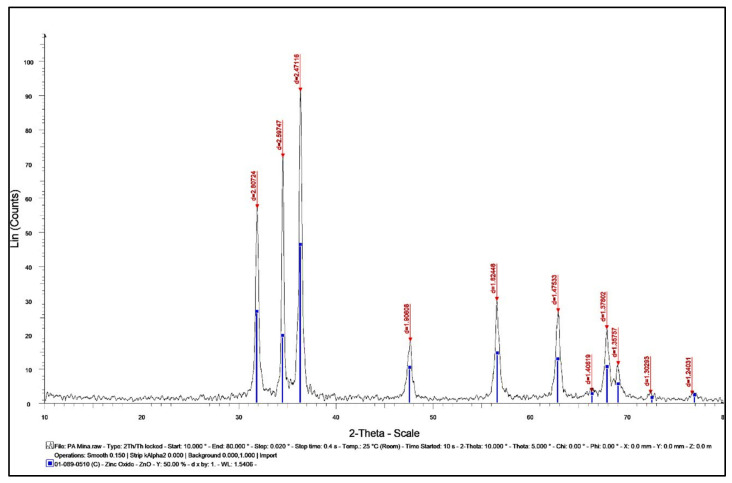
XRD analysis of ZnO NPs.

The existence of Nano-ZnO and exploration of their structural characteristics were confirmed by using X-ray diffraction (XRD). Nano-ZnO bio-synthesized by *P. auriculata* extract exhibited peaks with 2θ values existed at 31.852, 34.502, 36.325, 47.673, 56.613, 62.949, 66.431, 67.972, 69.14, 72.485, and 76.786 corresponding to (100), (002), (101), (102), (110), (103), (200), (112), (201), (004), (202), (104), and (203), respectively (Figure 10). Additionally, Nano-ZnO was considered to be pure due to the absence of characteristic XRD peaks other than the observed zinc oxide peaks [22,23]. These peaks were demonstrated in a crystal system of hexagonal phase with a space group of P63mc and reference code of (01-089-0510).

The average crystal size of Nano-ZnO was computed to be 35.34 nm. This data was very similar to the SEM measurement.

To calculate the crystal size, Scherrer’s equation was applied.

Crystal Size = (0.9 × λ)/(d cos θ) where Θ = 2θ/2, d = the full width at half maximum intensity of the peak (in Rad), and λ = 0.154060 nm.

### 2.3. The Cell Viability and Antiviral Activities of P. auriculata Lam. Extract and ZnO NPs

In vitro antiviral assessment investigated the virus’s capabilities for infection and multiplication at definite cell lines in culture systems. The cell culture system provided a rapid and accurate method to grow viruses at higher titers, produce vaccine strain cultures, study reverse genetics and inspect the antiviral compounds. Plants used in traditional medicine have been consumed for the treatment of various health circumstances, including infectious ailments. Nearly 60% of the anti-tumor and anti-infective remedies that are commercially available are derived from natural product origin. Therefore, traditional medicinal plants may serve as potent sources for acquiring new antiviral agents in the coming years. The acquiring of new antiviral drugs is a problematic mission due to the poor selective toxicity and the appearance of resistant viral variants that naturally arise. The occurrence of viral resistance to antiviral drugs is increasing and, consequently, viral diseases remain challenging to treat. The assessment of plants as promising origins for antiviral compounds has led to the detection of potent inhibitors of viral multiplication, increasing the possibility of detecting new bioactive plant compounds [24,25].

The genus *Plumbago* has previously been used for the treatment of various viral infections. The methanolic extract of *P*. *zeylanica* root showed antiviral activity against coxsackievirus B3 Nancy (CVB3), influenza A virus Hong Kong/1/68 (H3N2), and herpes simplex virus type 1 Kupka (HSV-1) at a concentration range of 0.8–200 μg/mL [26]. *P. indica* L. roots extracts showed remarkable inhibition of influenza A (H1N1) virus by inhibiting viral nucleoprotein synthesis and polymerase activity [27].

Metal and metal oxide NPs applications in virus-targeting formulations have shown the remarkable diagnostic or therapeutic ability of the agents, augmenting the targeted drug delivery functions [11]. ZnO NPs, due to their physiochemical characteristics, are considered a promising approach in developing antiviral agents and Nano-vaccines against RNA viruses HIV, and COVID19, as well as DNA viruses HSV-1 and 2. The most probable antiviral mechanistic pathways of ZnO NPs are blocking the virus’s entry into the cells and deactivating the virus through virostatic potential [28,29].

The pneumoviruses are composed of various human and animal pathogens such as human respiratory syncytial virus (hRSV), human metapneumovirus (hMPV), bovine respiratory syncytial virus (RSV), and avian metapneumovirus (aMPV). Among these viruses, aMPV is one of the leading causes of high economic losses in infected poultry. aMPV mainly causes infection of the upper respiratory tract in both chickens and turkeys, although turkeys seem to be more susceptible [30].

*P. auriculata* extract and biosynthesized ZnO nanoparticles were assessed for antiviral capabilities against aMPV subtype B. The results revealed that there are significant activities of *P. auriculata* extract and Nano-ZnO with SI more than 1.2. In antiviral analysis, it is necessary to define the maximum concentrations of the extract and ZnO NPs that is not toxic to the cells (MNTC). *P. auriculata* extract showed cytotoxic effect lower than Nano-ZnO with MNTC values equal to 235.4 and 173.5 μg mL^−1^, respectively. The CC_50_ for the extract and ZnO NPs were well tolerated by the CER cells. The CC_50_ for both the investigated agents was higher than 50 μg mL^−1^ and none of them produced any visible changes in cellular morphology or density. The CC_50_ and IC_50_ concentrations of both investigated agents found to be active against aMPV subtype B were calculated and recorded in Table 2. The screening of the antiviral performance of *P. auriculata* extract and ZnO NPs revealed that medium percentages of cell destruction were detected on CER cell line as they attained moderate antiviral inhibitory effects with CC_50_ of 87.00 ± 3.76 and 52.48 ± 1.57 μg/mL, respectively. Remarkable antiviral activity was observed for *P. auriculata* Lam. extract (67.97 ± 4.63 μg/mL) and Nano-ZnO (42.67 ± 4.08 μg /mL) for MOI of 0.001 ID_50_/cells. The antiviral activity of zinc oxide could be due to interference with the zinc binding activity of a viral protein, M2-1, that is found in all known pneumoviruses and is essential for virus replication and pathogenesis in vivo [30]. The antiviral activity of *P. auriculata* could be due to the chelating activity of phenolic compounds present in the extract for metal ions, especially zinc, that is essential for viral replication [30,31]. These results revealed that ZnO NPs have superior activity to the plant extract.

## 3. Materials and Methods

### 3.1. Plant Material

Flowering aerial parts of *P. auriculata* Lam. were acquired from EL-MAZHAR botanical garden, Giza, Egypt. The plant was kindly authenticated by Dr. Mohamed El Gebaly, Botany Taxonomist at the National Research Centre Herbarium, Dokki, Giza, and Engineered by Therease Labib, Consultant for plant identification at El-Orman botanical garden. Voucher specimen (numbered 16062020) was reserved at the herbarium of the Department of Pharmacognosy, Faculty of Pharmacy, Cairo University. *P. auriculata* was air-dried, coarsely powdered and kept in tightly closed, amber-colored glass containers at room temperature.

### 3.2. Extraction of Plant Active Ingredients

The air-dried powder of the flowering aerial parts of *P. auricolata* (100 g) was extracted using 90% ethanol (250 mL × 3). The extract was evaporated under reduced pressure using Buchi rotary evaporators until completely dry (2.5 g).

### 3.3. HPLC Analysis

HPLC analysis was performed by means of an Agilent 1260 series. The separation was performed by Eclipse C18 column (4.6 mm × 250 mm i.d., 5 μm). The mobile phase comprised (A) water and (B) 0.05% trifluoroacetic acid in acetonitrile using a flow rate 1 mL/min. The mobile phase was automatically flowed in a linear gradient as follows: 0 min (82% A and 18% B); 0–5 min (80% A and 20% B); 5–12 min (60% A and 40% B); and 12–16 min (82% A and 18% B). The detector used was multi-wavelength, adjusted to 280 nm. The injection volume was 10 μl for the standard and extract solutions. The column temperature was set at 35 °C.

### 3.4. Green Synthesis of ZnO NPs

ZnO NPs were synthesized using the alcoholic extract of flowering aerial parts of *P. auricolata* by a method illustrated by Attia et al. [32] with slight modification, in which *P. auriculata* Lam. dried extract (1 g) dissolved in ethanol (100 mL) was reacted with zinc acetate (10 g) dissolved in doubly distilled water (1000 mL) and heated in a boiling water bath for 20 min. Ammonium hydroxide (a few drops) was then added to the reaction to raise the pH to 12, where a Nano-ZnO precipitate was formed. The mixture was set for half an hour for complete reduction of zinc acetate to Nano-ZnO. The formed ZnO was centrifuged at 4000 rpm, followed by washing by bi-distilled water (two times), and ethanol (two washes) to yield white pellets of Nano-ZnO upon freeze drying.

### 3.5. Characterization of ZnO NPs

The Nano-ZnO was initially analyzed by means of a Shimadzu UV-1601 (Shimadzu Corporation, Japan) UV-vis spectroscopy instrument, ranging between 200 and 600 nm. Then by FTIR analysis using attenuated total reflectance (ATR) mode by a Jasco FTIR 4100 spectrophotometer (Japan) to categorize the functional groups and various phytochemical compounds responsible for formation and stabilization of the nanoparticles. Dynamic light scattering (DLS) analysis was performed using a Zetasizer (HT Laser, ZEN3600 Malvern Instruments, Malvern, UK) to investigate particle size and zeta potential of the prepared nanoparticles. Morphology and particle size of Nano-ZnO mediated by *P. auriculata* Lam. extract were determined by TEM (JEOL-JEM-1011, Japan) and FE-SEM (Mira3 Tescan). A few drops from the suspension of ZnO nanoparticles was applied on a carbon-coated copper grid and the solvent was evaporated at room temperature before recording the images. The powdered sample was subjected to CuKα1 X-ray diffractometer radiation (λ = 1.5406 A°) operating at 40 kV and 30 mA with 2θ ranging from 20°–90° to verify the occurrence of ZnO crystals and define their structure and size.

### 3.6. Antiviral Assessment

The antiviral activities of *P. auricolata* extract and ZnO NPs were investigated as described earlier according to Khon et al. [33] using chicken embryo related cells (CER cells) and aMPV strain UK/8/94 belonging to aMPV subtype B (GenBank Accession number Y14294). Maximum nontoxic concentrations (MNTC) for both investigated agents were determined and measured via the sulforhodamine B (SRB) assay [34]. Virus titers were determined by virus-produced cytopathic effect in cell culture and were labelled as 50% tissue culture infective concentration (TCID_50_) per mL. The TCID_50_ was computed according to Reed and Munch [35]. The inhibition percentage was calculated by using the formula: (IP) = (1 − T/C) × 100, where T is the antilog of the investigated agents-treated viral titers and C is the antilog of the control (without extract or Nano-ZnO) viral titers. IP was estimated to be positive if greater than or equal to 98%. The 50% cytotoxic (CC_50_) and 50% inhibition (IC_50_) concentrations for the plant extract and Nano-ZnO were computed from their concentration–response curves obtained by different concentrations for both investigated agents in the presence of 100 TCID_50_/mL using the MTT assay [36,37]. The selective index (SI) was computed from a ratio of CC_50_/IC_50._ The results were acquired from triplicate assays with a minimum of five concentrations for *P. auricolata* extract and nanoparticles. The cytotoxicity percentage was obtained from [(A1 − A2)/A1] × 100, where A1 and A2 are the OD_540_ nm of untreated and of treated cells, respectively. The protection percentages were computed from [(X1 − X2) × 100/ (X3 − X2)], where X1, X2, and X3 stand for the absorbance values of the extracts or ZnO NPs, virus, and cell controls, respectively.

## 4. Conclusions

This current work aimed to biosynthesize ZnO NPs from *P. auriculata* alcoholic extract and investigate the antiviral effects of the plant extracts and ZnO nanoparticles against aMPV subtype B. From this study, it can be speculated that the engagement of *P. auriculata* extract and/or ZnO nanoparticles with vaccination would be beneficial for the control of virus spreading in infected birds. In societies where this disease is endemic, this will reduce the environmental contamination and consequently the risk of virus spreading. Our in vitro results suggest that supplementing the poultry diet with *P. auriculata* extract and ZnO nanoparticles could be useful for controlling enteric infections of aMPV; however, further in vivo trials are required to confirm the antiviral activity observed in vitro and to find the optimum concentration of *P. auriculata* and Nano-ZnO required in bird diets to have antiviral effectiveness due to poultry digestive tract complexity.

## Figures and Tables

**Table 1:** Phenolic compounds of *P. auriculata* Lam. aerial parts identified by HPLC.

	RT ^1^	RRT ^2^	Area *	Area%	Conc. (µg/g)
Gallic acid	3.032	0.43	533.43 ± 2.3	21.20	1720.26
Chlorogenic acid	3.642	0.52	585.81 ± 1.07	23.20	1600.42
Catechin	4.058	0.58	193.40 ± 2.31	7.60	840.20
Methyl gallate	4.992	0.71	58.45 ± 0.84	2.36	29.57
Coffeic acid	5.302	0.75	56.58 ± 0.71	2.21	87.04
Syringic acid	5.888	0.84	150.87 ± 1.38	6.03	243.48
Pyro catechol	6.148	0.87	38.71 ± 0.49	1.50	97.97
Rutin	7.034	1.00	99.781 ± 1.03	3.96	497.51
Ellagic acid	7.694	1.09	30.84 ± 3.66	12.05	744.63
Coumaric acid	8.231	1.17	93.67 ± 2.13	3.68	57.69
Vanillin	8.857	1.26	42.39 ± 1.06	1.64	34.07
Ferulic acid	9.616	1.37	41.03 ± 0.86	1.59	51.14
Naringenin	10.108	1.44	142.74 ± 1.51	5.70	291.09
Taxifolin	11.897	1.69	25.49 ± 0.64	1.01	67.83
Cinnamic acid	13.022	1.85	58.95 ± 0.42	2.38	22.18
Kaempferol	14.128	2.01	97.23 ± 0.26	3.90	141.09

^1^ RT, retention time; ^2^ RRT, relative retention time to rutin.; * Area expressed as mean ± S.E.

**Table 2:** Antiviral activity of *P. auriculata* extract and ZnO NPs against aMPV subtype B.

	I.P	CC_50_ μg/mL *	IC_50_ μg/mL *	SI
*P. auriculata*	99%	87.00 ± 3.76	67.97 ± 4.63	1.28
ZnO NPs	99%	52.48 ± 1.57	42.67 ± 4.08	1.23

I.P, inhibition percentage; CC_50_, 50% cytotoxic concentration; IC_50_, 50% inhibition concentration; SI, selective index. * Values are expressed as mean ± S.E.

## Data Availability

Not applicable.
